# The Effect of Hydrogen Content on Ballistic Transport Behaviors in the Ni-Nb-Zr-H Glassy Alloys

**DOI:** 10.3390/ijms13010180

**Published:** 2011-12-23

**Authors:** Mikio Fukuhara, Yoshimasa Umemori

**Affiliations:** Institute for Materials Research, Tohoku University, Sendai 980-8577, Japan; E-Mail: umemori@imr.tohoku.ac.jp

**Keywords:** glassy alloy, centimeter-sized ballistic transport, hydrogen doping, icosahedral cluster

## Abstract

The electronic transport behaviors of (Ni_0.39_Nb_0.25_Zr_0.35_)_100−_*_x_*H*_x_* (0 ≤ *x* < 23.5) glassy alloys with subnanostructural icosahedral Zr_5_Nb_5_Ni_3_ clusters have been studied as a function of hydrogen content. These alloys show semiconducting, electric current-induced voltage (Coulomb) oscillation and ballistic transport behaviors. Coulomb oscillation and ballistic transport occur at hydrogen contents between 6.7 and 13.5 at% and between 13.5 and 21.2 at%, respectively. These results suggest that the localization effect of hydrogen in the clusters plays an important role in various electron transport phenomena.

## 1. Introduction

Glassy alloys are peculiar metallic alloys because they lack, on the nanoscale, the long-range translational order of crystalline alloys [1]. Therefore, much attention has been devoted to examining their diverse electronic characteristics. In particular, we have found that (Ni_0.39_Nb_0.25_Zr_0.35_)_100−_*_x_*H*_x_* (0 < *x* < 20) alloys are characterized by semi-, superior (ballistic)- and superconductivity, and these alloys show electron avalanche and Coulomb oscillation at higher hydrogen content [2]. Following the discovery of centimeter-sized ballistic conductivity, we have studied a rotating speed effect on electronic transport behaviors of Ni-Nb-Zr-H glassy alloys. Supercooling (104.7-m/s) of the molten alloy produces a ballistic conductor with electrical conductivity of about 0.1 nΩ·cm (0.01% of silver (1.62 μΩ·cm)) for (Ni_0.39_Nb_0.26_Zr_0.35_)_97.8_H_2.2_ glassy alloy and a room-temperature discrete Coulomb oscillation for (Ni_0.39_Nb_0.25_Zr_0.35_)_95.2_H_4.8_ glassy alloy [3]. The increase in degree of amorphousness by supercooling induces uniformity of cluster morphology, leading to superior conductivity and quantization of Coulomb oscillation. We postulated the existence of macroscopic quantum electron tunnels passing along the millimeter-sized zigzag paths of atomic bond arrays with a large capacitance (of the order of several femtofarad) among Ni-centered ideal [4] and Zr-centered distorted [3,5] icosahedral Zr_5_Ni_5_Nb_3_ clusters for ballistic transport and Coulomb oscillation, respectively, although the amorphous structure in glassy alloys is composed of a large number of low symmetry-clusters located around the main icosahedral ones.

In this study, we report the effect of hydrogen content on ballistic transport behaviors in the Ni_39_Nb_25_Zr_35_ glassy alloys with subnanometer-scale sized clusters, as a representative composition for ballistic behavior. This superior conducting behavior resembles the ballistic transport observed in one-dimensional, nanometer-scale channels, such as quantum wires [6], carbon nanotubes [7,8] and GaAs-AlGaAs [9] at low temperature, in the form of quantum interference associated with coherence. The ballistic electron transport effect is promising for future electron devices and electric power applications such as lower supply voltage and leading to low power consumption. However, no research work has been carried out on this subject for glassy alloys with hydrogen, as far as we know.

## 2. Experimental

The rotating wheel method under a helium atmosphere was used for preparing amorphous Ni_39_Nb_25_Zr_35_ alloy ribbons of 1-mm width and 20-μm thickness, using of rotation speed of 6000-rpm (62.8-m/s). Hydrogen charging was carried out electrolytically in 0.5 M H_2_SO_4_ and 1.4 g/L thiourea (H_2_NCSNH_2_) at room temperature and current densities of 30 A/m^2^ [10–13]. The charged specimens passed one week after charging were used in order to prevent inhomogeneous distribution of hydrogen. The amounts of hydrogen absorbed in the specimens were measured by the inert gas carrier melting-thermal conductivity method. The structure of the glassy alloy was identified by X-ray diffraction with Cu Kα radiation in the grazing incident mode.

The specific electrical resistance of hydrogenated specimens was measured by the four-probe method DC and AC Current Source 6221, Nano Voltmeter 2182A (Keithley Instruments Inc.) with a dc current of ±1 mA at cooling and heating rates of 0.017 K/s from 373 K to 6 K in He of ambient pressure (Top Loading Refrigerated Cryostat; JECC Torisha Co.) [2–5,9–13]. The distance between the two voltage electrodes was 20 mm. To make clear hydrogen inhomogeneous effect, we also measured resistivity after polishing on both sides by 5 μm.

## 3. Results and Discussion

Visibly, all the ribbons prepared by rotating wheel method were mirror-like and showed good toughness. Change from argon to helium atmosphere gasses makes the surface smooth due to one-order higher thermal conductivity of helium (Ar: 0.1772 W/(m K), He: 0.152 W/(m K) [14]). This material showed three kinds of transport behaviors, semi-conducting, Coulomb oscillation and ballistic transport behaviors.

**Figure 1:**
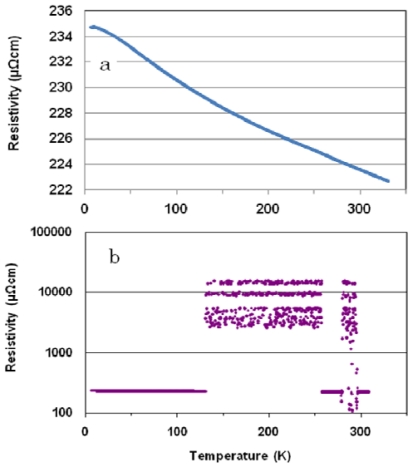
The temperature dependence of the resistivity of polished (Ni_0.39_Nb_0.26_Zr_0.35_)_90.5_H_9.5_ glassy alloy in cooling run (**a**) and heating run (**b**).

For a cooling run of the polished (Ni_0.39_Nb_0.26_Zr_0.35_)_90.5_H_9.5_ glassy alloy, the resistivity (Figure 1a) shows a sluggish increase from 300 K down to 6.5 K (similar to a semiconducting material). The same trend is also shown for a heating run up to 163 K in (Figure 1b). However, the resistivity shows current-induced discrete oscillations in a temperature region of between 163 to 295 K. Quantization of Coulomb oscillation is associated with a high number of subnanometer-sized RC circuits constructed by a pair of pointed Zr_5_Ni_5_Nb_3_ cluster channels and perpendicularly connecting cluster array reservoirs that contain subnanometer-sized capacitors in the alloy [13]. It has already been reported that the Coulomb oscillation is periodic [10–12].

**Figure 2:**
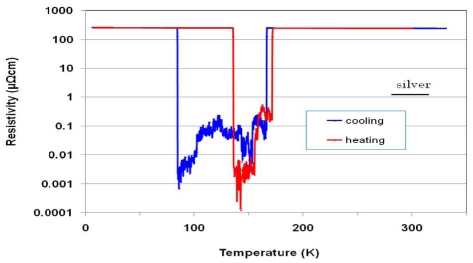
The temperature dependence of the resistivity of polished (Ni_0.39_Nb_0.26_Zr_0.35_)_85_H_15_ glassy alloy in cooling run (**a**) and heating run (**b**).

When hydrogen content increases over 13.5 at%, ballistic transport behavior occurs. Figure 2 is a representative example for polished (Ni_0.39_Nb_0.26_Zr_0.35_)_85_H_15_ glassy alloy. In cooling run, the resistivity decreases almost linearly down to 170 K and then suddenly reduces by three orders of magnitude at 165 K and falls down again to order of 0.001 μΩ·cm at 88 K. Subsequently the resistivity recovers abruptly on the extended line of the cooling curve between 330 and 170 K, and then ascends once again as temperature decreases. In the heating run, the resistivity decreased according to the same curve as the cooling run, except for a drop down to order of 0.001 μΩ·cm between 130 and 172 K. The resistivity (0.1 nΩ·cm) at 130 K in heating run is around 0.006% of silver (1.62 μΩ·cm) at room temperature.

**Figure 3:**
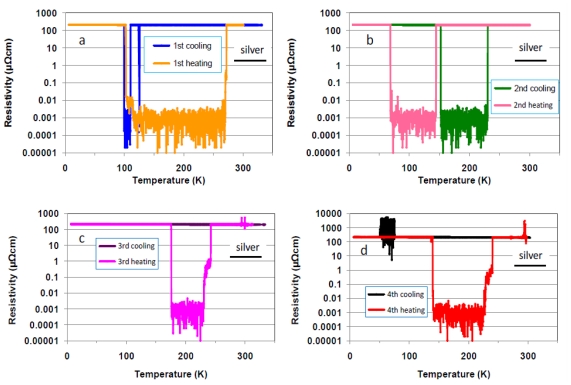
The temperature dependence of the resistivity of polished (Ni_0.39_Nb_0.26_Zr_0.35_)_78.8_H_21.2_ glassy alloy in the 1st cooling and heating runs (**a**); the 2nd runs (**b**); the 3rd runs (**c**); and the 4th runs (**d**).

To ensure the reproducibility of these changes of the resistance and the domain of existence of the ballistic transport effect, we repeated the cooling and heating runs four times—measurement of ballistic transport behavior in a temperature region of between 300 and 6 K—using the polished (Ni_0.39_Nb_0.26_Zr_0.35_)_78.8_H_21.2_ glassy alloy. The 2nd and 4th runs followed repeatedly the 1st and 3rd runs, respectively, but the 3rd runs were carried out after 5 days of the 2nd runs. The ballistic transport occurs in both cooling and heating runs for the 1st and 2nd runs, but in the heating run alone for the 3rd and 4th runs. Since a metal/ballistic transition occurs within narrow temperature region, the transition would be derived from morphology changes associated with two types of cluster ordering: topological ordering and compositional short-range orderings [15]. As can be seen from Figures 2 and 3, the ballistic transport occurs in a temperature region of between 65 and 230 K, but there is no regular rule for holding temperature region. Therefore, the increase in degree of amorphousness, *i.e*., supercooling of the molten alloy will help uniformity of cluster morphology [3], leading to occurrence of ballistic transport up to 272 K. Since it is assumed that the mean free path of electrons in a glassy alloy is much larger than the width of the tunnels (0.23 nm [5]) between the clusters, we can imagine the existence of macroscopic quantum electron tunnels passing along the arrays. Furthermore, the Coulomb oscillation, which is observed for alloy with lower hydrogen content (in Figure 1b), revealed in lower temperature region of the 4th cooling run. For this reason, we assume that repeated cooling/heating runs induces formation of capacitance tunnels with femtofarad capacitance among clusters.

**Figure 4:**
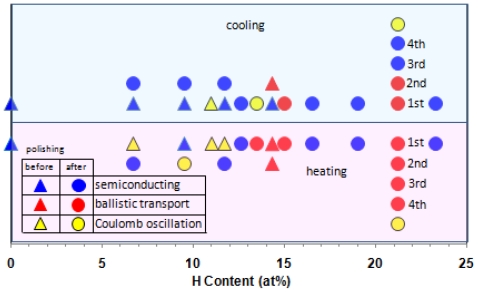
The effect of H content on the occurrence of semiconducting, ballistic transport and Coulomb oscillation properties of the (Ni_0.39_Nb_0.25_Zr_0.35_)_100−_*_x_*H*_x_* (0 ≤ *x* < 23.5) glassy alloys.

We summarize electronic transport behaviors for cooling and heating runs of the hydrogenated (Ni_0.39_Nb_0.25_Zr_0.35_)_100−_*_x_*H*_x_* (0 ≤ *x* < 23.5) glassy alloys before and after polishing (Figure 4). The glassy alloys display three-kinds of electronic transport behaviors, semiconducting, ballistic transports and Coulomb oscillation. The Coulomb oscillation occurs below hydrogen content of 13.5 at% except for 21.2 at% H-specimen under repeated cooling and heating runs, and the ballistic transport appears at hydrogen content region of between 13.5 and 21.2 at%. This trend is similar to previous result of hydrogenated (Ni_0.39_Nb_0.25_Zr_0.35_)_100−_*_x_*H*_x_* (0 < *x* < 20) glassy alloys obtained by rotating speed of 3000 rpm (31.4 m/s) [2]. In addition, we observed the stability of the resistance with the time at a constant temperature in the region characterized by these Coulomb oscillation and ballistic transport phenomena. Furthermore, we observed similar behaviors on ribbons prepared from different batches.

From these results, we assume that for Coulomb oscillation, the inhomogeneous hydrogen absorption below hydrogen content of 13.5 at% in Ni-centered ideal icosahedral clusters facilitates formation of subnanometer scaled capacitance tunnels, and for ballistic transport, full hydrogen absorption into the clusters in hydrogen content between 13.5 and 21.2 at% assists regular array formation of clusters. The ballistic transport and Coulomb oscillation prefer to occur in heating run rather than in cooling run. This suggests that occurrence of both phenomena needs higher degree of amorphousness, *i.e*., uniformity of cluster morphology. Furthermore, we found a similar resistance result for electronic transport behaviors of the hydrogenated glassy alloys before and after polishing. This indicates that there is no inhomogeneous distribution effect of hydrogen.

## 4. Conclusions

Characteristic electric resistivity of the (Ni_0.39_Nb_0.25_Zr_0.35_)_100−_*_x_*H*_x_* (0 ≤ *x* < 23.5) glassy alloys with Ni-centered subnanostructural icosahedral Zr_5_Nb_5_Ni_3_ clusters was measured in a temperature region of between 330 and 6 K. The glassy alloys display three-kinds of electronic transport behaviors, semiconducting, ballistic transports and Coulomb oscillation. The resistivity of the (Ni_0.39_Nb_0.25_Zr_0.35_)_100−_*_x_*H*_x_* (6.7 < *x* < 13.5) alloys showed the electric current-induced voltage (Coulomb) oscillation. The hydrogenated (Ni_0.39_Nb_0.25_Zr_0.35_)_100−_*_x_*H*_x_* alloy (13.5 < *x* < 21.2) revealed ballistic transport in a temperature region of between 65 and 230 K, which was around 0.006% of resistivity of silver at room temperature. However, there was no regular rule for holding the temperature region. From these results, we assume that for Coulomb oscillation, the inhomogeneous hydrogen absorption below hydrogen content of 13.5 at% in Ni-centered ideal icosahedral clusters, facilitates formation of subnanometer scaled capacitance tunnels on the outer space of the clusters; and for ballistic transport, full hydrogen absorption into the clusters in hydrogen content between 13.5 and 21.2 at%, assists regular array formation of clusters. Thus this paper will throw new light for innovative technologies based on cluster science.
